# Agenesis of maxillary lateral incisors: diagnosis and treatment options

**DOI:** 10.1590/2177-6709.27.1.e22spe1

**Published:** 2022-06-06

**Authors:** Daniela Kimaid SCHROEDER, Marco Antonio SCHROEDER, Viviane VASCONCELOS

**Affiliations:** 1 Private practice (Rio de Janeiro/RJ, Brazil).; 2 Private practice (Madrid, Spain).

**Keywords:** Congenital absence, Maxillary lateral incisors, Smile esthetics, Orthodontic planning, Canine guidance, Group function

## Abstract

**Introduction:**

There are different possibilities of orthodontic planning for cases with congenital absence of maxillary lateral incisors. This subject divides the opinion of orthodontists and oral rehabilitation clinicians, due to the advantages and disadvantages of each treatment option, which may involve opening spaces for future implants and/or prosthetic restorations, or closing the spaces by positioning the maxillary canines in the place of lateral incisors. The correct diagnosis and careful evaluation of each patient allow to determine the best therapeutic approach. This paper discusses the main topics to be considered when planning these cases.

**Objectives:**

To evaluate the main aspects related to orthodontic treatment planning in cases of congenital absence of maxillary lateral incisors, to aid the decision-making, with clinical and scientific basis.

## RESUMO

**Introdução:**

Existem diferentes possibilidades de planejamento ortodôntico para os casos que apresentam ausência congênita de incisivos laterais superiores. Esse é um assunto que divide a opinião de ortodontistas e reabilitadores orais, devido às vantagens e desvantagens de cada uma das opções de tratamento, as quais podem envolver a abertura de espaços para futuros implantes e/ou restaurações protéticas ou o fechamento dos espaços, com posicionamento dos caninos superiores no lugar dos incisivos laterais. O correto diagnóstico e uma criteriosa avaliação de cada paciente permitem determinar a melhor abordagem terapêutica. Nesse artigo, serão discutidos os principais tópicos a serem considerados no planejamento desses casos.

**Objetivos:**

Avaliar os principais aspectos relacionados ao planejamento do tratamento ortodôntico nos casos de ausência congênita de incisivos laterais superiores, de maneira a auxiliar nas tomadas de decisão, com embasamento clínico e científico.

## INTRODUCTION

The hypodontia of one or more permanent teeth is one of the most common developmental anomalies in humans. A tooth is defined as congenitally absent when it has not erupted into the oral cavity, has not been extracted or accidentally lost, and is not visible on radiographic examination.^1^ The etiopathogenesis is not fully understood, yet there is evidence of environmental and inherited causes or even the interaction between them.

Hypodontia occurs mainly in Caucasian patients and women. The third molars are the most affected, followed by mandibular second premolars, maxillary lateral incisors and maxillary second premolars.^2^

There are different possibilities of orthodontic treatment for cases with congenital unilateral or bilateral absence of the maxillary lateral incisors. This subject divides the opinion of orthodontists and oral rehabilitators, due to the advantages and disadvantages of each treatment option, which may include opening spaces for future implants and/or prosthetic restorations, or closing spaces by positioning the maxillary canines in the place of lateral incisors.

Regardless of the treatment chosen, the main objective of the orthodontist is to achieve a satisfactory and stable esthetic and functional result, with long-term stability. Many challenges are involved in this process, and a careful assessment must be done before decision making.

Factors as patient age, type of malocclusion, relationship of anterior teeth, facial profile, size, shape and shade of canines, as well as smile line height, should be considered.

The main criteria to be observed and considered in the diagnosis and planning of cases with congenital absence of maxillary lateral incisors will be discussed below.

## PATIENT AGE

**Figure 1:**
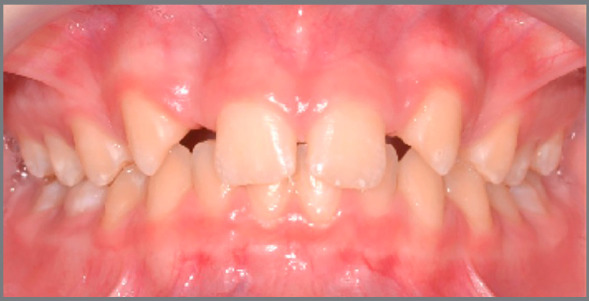
11-year-old female patient with esthetic complaint due to absence of maxillary lateral incisors.

**Figure 2:**
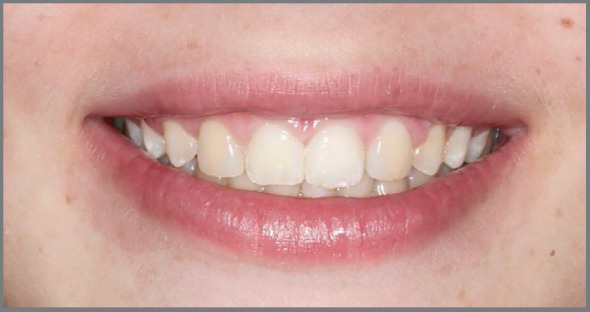
Smile at 13 years of age. Case treated with space closure, with maxillary canines replacing the missing maxillary lateral incisors.

This is a relevant and maybe the main factor in decision making. Currently, orthodontists commonly receive patients with absence of maxillary lateral incisors at a very young age, with esthetic complaint (Fig 1). Usually, the permanent canines have erupted in a more mesial position, filling part of the lateral incisors space, which already simplifies the orthodontic closure of spaces. The psychological pressure that this patient may undergo due to impaired esthetics is also a matter of concern for parents and orthodontists. This fact influences the decision for orthodontic space closure, since the patient will be able to spend the remaining adolescence period with the treatment already completed, with the natural dentition and without esthetic problems (Fig 2).^3^

This approach allows maintenance of dental and periodontal architecture in their natural state, with better response to changes over time.

The option of opening spaces for implants at an early age must consider the risk of loss of alveolar bone height and thickness, since the implants can only be placed after growth completion. The need for provisional prostheses until implant placement causes a waiting period, which is a reason for frequent patient dissatisfaction. Combined to this, there is possibility of inclination of roots of teeth adjacent to the space, requiring retreatment in the future.^4^

In young patients, it is not possible to predict the tissue response around the implants. For this reason, when planning includes space opening, Zachrisson et al.^5^ suggested the posterior regions, preferably in premolars region.

One disadvantage of this type of approach is the possible reopening of spaces after treatment completion. To avoid this, it is recommended to use a fixed retainer bonded on the palatal aspect of maxillary anterior teeth, after restorative esthetic recontouring of the anterior teeth.

**Figure 3:**
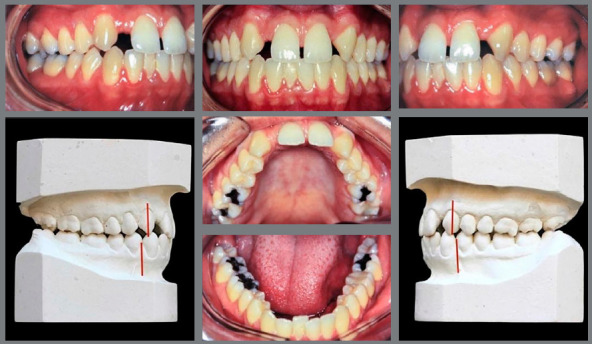
Adult patient, with absence of maxillary lateral incisors, maxillary anterior spaces and mandibular crowding. Class II canine relationship, mild overbite and overjet.

**Figure 4:**
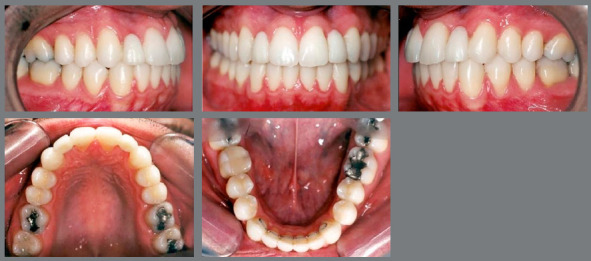
Case treated with space opening for replacement of maxillary lateral incisors with dental implants.

Concerning adult patients (Fig 3), decision-making should be based mainly on the anteroposterior relationship of teeth, smile line height and patient profile. If there is no significant facial and dentoalveolar growth, the orthodontist may provide excellent results in shorter treatment periods (Fig 4).

## INITIAL MALOCCLUSION AND PROFILE

Several authors agree that the ideal dentofacial patterns for space closure involve patients with Angle Class II malocclusion, balanced profile and absence of crowding in the mandibular arch.^6^ ^,^ ^7^

**Figure 5:**
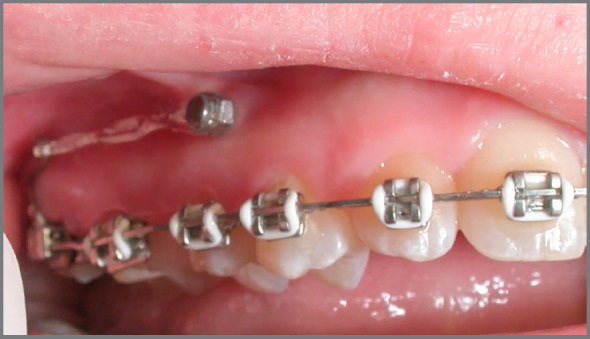
Hook made with stainless steel wire, fitted to the distal part of the tube of tooth #17, connected to the mini-implant between the roots of teeth #14 and #15 using a chain elastic, aiming at mesialization of the posterior segment, closing the spaces by anchorage loss.

Mesial movement of the maxillary teeth can affect the axial inclination of maxillary incisors, making them excessively upright. This impairs the overbite and overjet (Figs 7C, 7D, 9A and 9B), as well as the relationship of maxillary incisors with the lips and profile. However, skeletal anchorage and the association with intermaxillary elastics aid the process of maxillary dentoalveolar mesialization, consequently achieving esthetic and functional occlusion, without damage to the profile, even in patients with maxillary deficiency.^8^ With the same objective, skeletal anchorage can also be used in the maxillary arch to lose anchorage of posterior teeth without altering the inclination of maxillary incisors, as shown in Figure 5.

## SMILE ANALYSIS

Patients who expose their gingiva when smiling may be harmed by replacing missing teeth with endosseous implants. Even with an excellent post-implant esthetic result, it may not be maintained over time. Over the years, the gingival and incisal margins tend to become uneven, leaving the implanted units in infraocclusion, since they do not follow the passive eruption process of adjacent teeth. This fact can interfere, less or more significantly, in the long-term smile esthetics.^6^

Implant placement in esthetic areas is a procedure that depends on the operator’s sensitivity, with little condition to predict the biological and technical complications that involve the procedure.

Deficiency of interdental papillae, gingival discoloration and, over the years, loss of bone height accompanied by increased probing depth can impair the outcome of orthodontic/rehabilitative treatment.^6^ ^,^ ^9^ ^,^ ^10^

**Figure 6:**
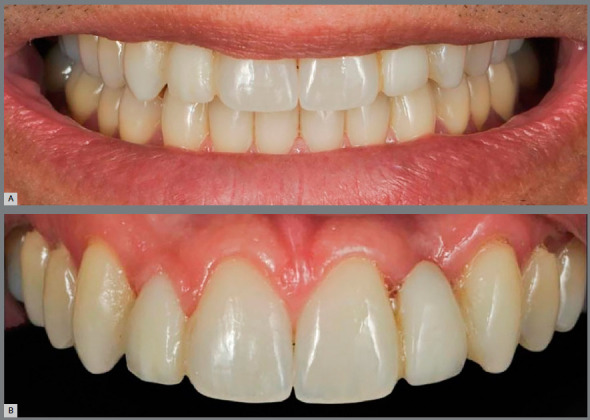
A) Image of adult patient with low smile, after orthodontic space opening for implant placement in the regions of maxillary lateral incisors. B) The presence of bone dehiscence on the buccal walls of teeth #12 and #22 could compromise the smile esthetics, if there was any degree of gingival exposure.

Conversely, adult patients with a low smile height, i.e., without any gingival exposure, may benefit from the option of implants replacing the maxillary lateral incisors (Fig 6).

## PERIODONTAL, OCCLUSAL AND JOINT HEALTH

**Figure 7:**
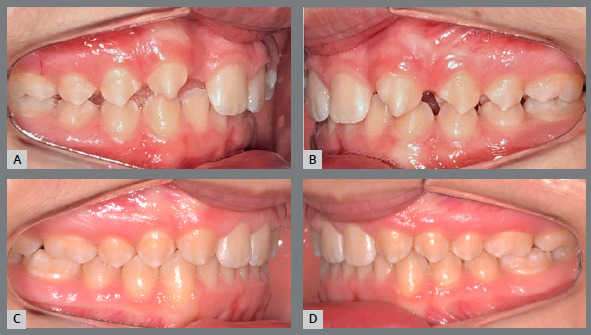
A, B) Lateral intraoral photographs of initial occlusion, with molars and canines in Class II relationship, congenital absence of maxillary lateral incisors, generalized spaces in the maxillary arch. C, D) Post-orthodontic treatment photographs showing closure of spaces, with mesialization of maxillary posterior teeth. Teeth #13 and #23 replaced the missing teeth #12 and #22, and the molar relationship settled into a full Class II relationship.

**Figure 8:**
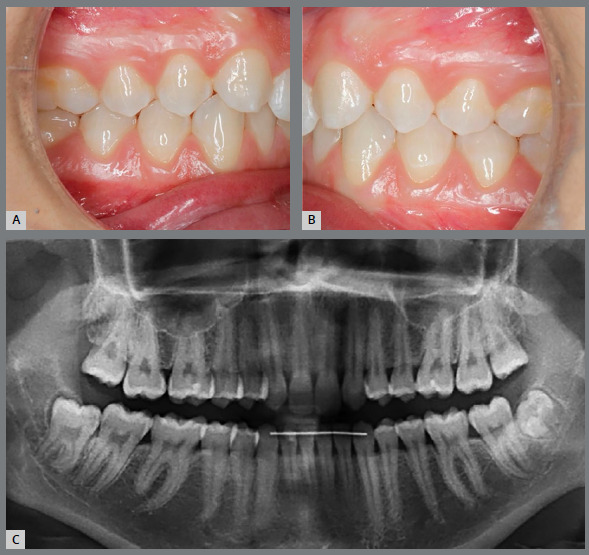
A, B) Posterior occlusion on the right and left sides, periodontal health in the premolar region with 7-year follow-up. C) Follow-up panoramic radiograph.

The periodontal status and aspects that influence the temporomandibular joints are other concerns that should be addressed. Some retrospective studies^11^ ^,^ ^12^ have shown that the periodontal health of patients treated with space closure is significantly better than patients who received prosthetic replacement of lateral incisors, and there are no differences in the adequacy of occlusal function between the two treatment options, as well as signs and symptoms of temporomandibular disorders (Figs 7 and 8).

According to Amm et al.^8^, the presence or absence of a canine occlusion key did not show correlation with occlusal function or signs and symptoms of temporomandibular disorder. This was also mentioned by Rosa et al.^19^ in a ten-year longitudinal follow-up study that reported on patients with maxillary lateral incisors agenesis treated with canine replacement. All had healthy and stable periodontal tissues, without attachment loss in the bone crests.^19^

In a systematic review conducted by Silveira et al.^13^ in 2016, the authors concluded that the presence or absence of canine relationship in occlusion was not related to occlusal function or with signs and symptoms of temporomandibular disorders.

Some authors have associated the lack of lateral disocclusion guidance in canines with high incidence of abfractions in the cervical regions of maxillary first premolars, which can be considered a disadvantage in the option of space closure.^14^ However, it is important to emphasize that the presence of non-carious cervical lesions has a multifactorial etiology and depends on the association of factors such as stress and friction, and the literature has weak convincing evidence indicating the superiority of lateral canine guidance in relation to group function.^15^ The systematic review performed by Abduo et al.^16^ demonstrates that there is no gold standard between the two types of disocclusion for all patients. According to the authors, both situations are equally acceptable. Also, they point out that young people tend to have canine guidances, and older individuals tend to present lateral group function, since the occlusion is dynamic, adaptable and subject to changes over time.^16^ ^,^ ^17^ ^,^ ^18^

**Figure 9:**
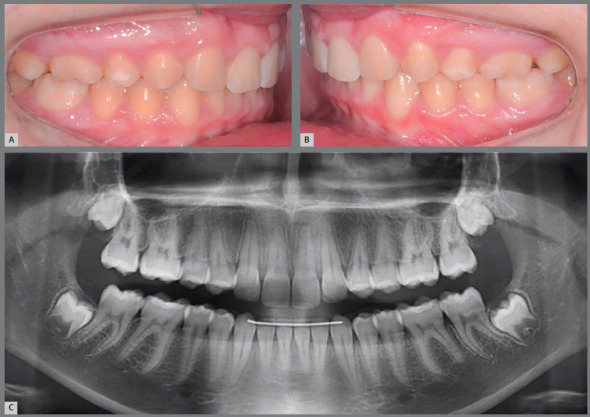
A, B) Occlusion after completion of orthodontic treatment, performed by replacing the congenitally absent maxillary lateral incisors with maxillary canines. C) Panoramic radiograph at treatment completion.

**Figure 10:**
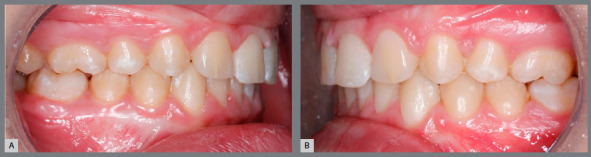
Photographs taken five years after treatment completion, showing a stable occlusion, besides dental and periodontal health.

**Figure 11:**
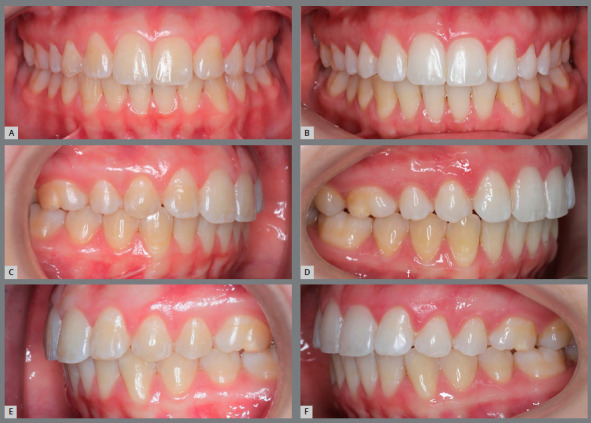
A) Frontal view of a case of congenital absence of teeth #12 and #22 orthodontically treated by space closure. B) Ten years after treatment completion, the patient started the tooth whitening process. C) Right lateral view of the final occlusion. D) Image ten years after treatment. E) Left lateral view of the final occlusion. F) The same image after ten years of treatment, evidencing on the photographs taken in the post-retention phase that the periodontium and anatomy of premolars involved with the group lateral disocclusion guidance remained healthy.

Another question refers to the ability of first premolars to withstand the occlusal forces that ideally occur on the canines, without mobility. In the studies by Rosa et al.^19^, Nordquist et al.^11^ and Robertsson et al.^12^, no statistically significant differences were found in the insertion of first premolars, showing that they can play an adequate functional occlusion in group disocclusions, without periodontal damage (Figs. 9, 10, 11 and 14).

To date, there is no consistent scientific evidence to justify the constant search for canine protected occlusion as beneficial for all orthodontic patients, especially those with missing maxillary lateral incisors.^17^

According to Silveira and Mucha^20^, the canine disocclusion guidance is not an absolute condition to maintain the balance of the stomatognathic system, since it can be replaced by group function. This simplistic approach is not supported by scientific evidence, but rather by dogmatic beliefs.^9^ ^,^ ^10^ ^,^ ^13^ ^,^ ^17^ ^,^ ^20^

## CANINE SHAPE AND SHADE

The anatomy of canines differs considerably from the anatomy of lateral incisors. They have larger mesiodistal and cervico-occlusal diameters, their buccal surfaces are convex, and they have a cusp. In most cases treated by closing spaces of the maxillary lateral incisors, it is necessary to re-shape the teeth #13 and #23, with wear on the cusp and palate and addition of restorative material on the mesial and distal surfaces, to adjust the esthetics and function.^10^ Additionally, their color is more yellow than that of its neighboring teeth. Although there are exceptions, the canine shade can be closer to the color of adjacent teeth when compared to the color of porcelain crowns. If this factor compromises the esthetics, individual bleaching can provide improvement, or even the use of resin restorations and/or porcelain veneers on anterior teeth.

More attention should still be dedicated to patients with unilateral agenesis, since there are commonly significant differences in size, shape and color between the lateral incisor and the canine on the opposite side. The lack of symmetry is perceived not only by dentists, but also by lay people.^21^ To make the dental arch more symmetrical, the need and possibility of mesiodistal increase of the present lateral incisor must be assessed.^11^ ^,^ ^23^ ^,^ ^24^ ^-^ ^27^

## FINISHING

**Figure 12:**
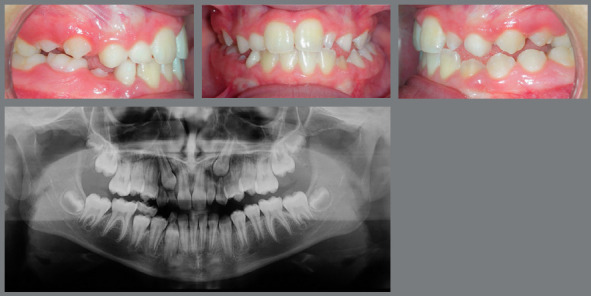
Young patient, aged 9 years at completion of mixed dentition, with molar and canine relationship in key occlusion, posterior crossbite on the left side, absence of tooth #12 and microdontia associated with a peg-shaped crown of tooth #22. The maxillary left canine had a radiographic appearance of possible impaction.

**Figure 13:**
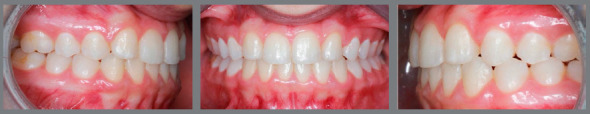
Images of occlusion after orthodontic treatment performed by closing the maxillary spaces. Expansion of maxillary arch and extraction of the peg-shaped lateral incisor were performed. Anatomical re-shaping was performed on the permanent maxillary canines, aiming at improving the esthetics and function.

**Figure 14:**
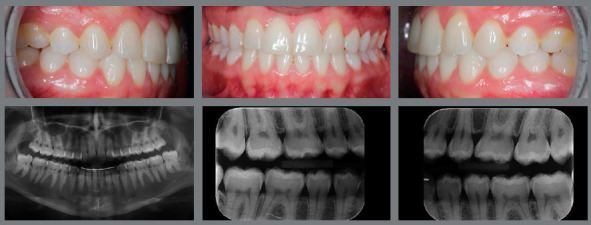
Images of the case, 8 years after completion of orthodontic treatment. Esthetic completion of the case was performed by restorative dentistry, by re-shaping of maxillary incisors, canines and first premolars.

**Figure 15:**
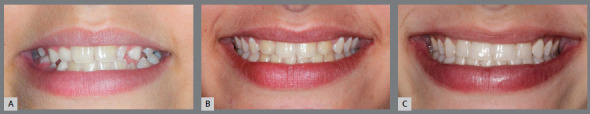
Smile images: A) Initial; B) After completion of orthodontic treatment, in which it is possible to observe the change in color and anatomy of canines; C) After esthetic restorative procedures.

According to some authors, the option of orthodontic space closure represents the most conservative solution. When the case planning includes the participation of restorative specialties, the treatment result usually presents excellent esthetic and functional results (Figs. 12, 13, 14 and 15).^9^ ^,^ ^10^ ^,^ ^23^ ^,^ ^24^

Restorative esthetic finishing resources involve the re-shaping of canines to improve the harmony and function.^3^ Another tooth to be evaluated at completion of treatment with space closure is the maxillary first premolar, which is usually shorter and narrower than the canine. Such discrepancies should be compensated with restorative and periodontal procedures, and the patients should be aware of this demand since the planning is discussed. It is common to find the need to also increase the central incisors in height and width, due to their reduced size, to obtain greater exposure of incisors during speech and smile.

Regardless of the plan chosen for each patient, the orthodontist needs to know how to work together with other specialties to achieve optimal results. In situations where the orthodontist decides to close the maxillary spaces, the participation of restorative dentistry and periodontics is nearly mandatory.^25^ ^,^ ^26^ ^,^ ^27^

Some orthodontic finishing details should be addressed when cases are treated with space closure. To achieve the best esthetic and functional result, the canine torque should be as similar as possible to that of the lateral incisor. The movement of its root in palatal direction reduces the alveolar bone eminence, improves the interproximal contact with the central incisor, and decreases the occlusal load with mandibular incisors during protrusive movement.

**Figure 16:**
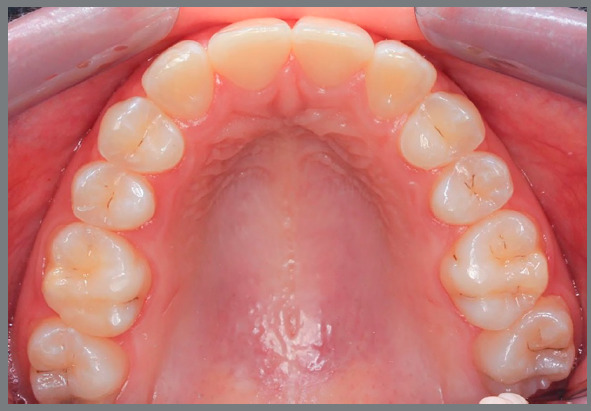
Canines with palatal root torque and slightly mesially rotated maxillary first premolars.

To allow the first premolar to resemble the canine in positioning, Tuverson^23^ suggests an offset for this tooth. A slight mesial rotation is recommended for the crown of the first premolar (Fig 16), which would eliminate interferences on the working and balance side, and reduce the need for cusp wear.^28^

When possible, custom intrusion of the first premolar and extrusion of the canine to correct the flatness of gingival margins is also indicated, aiming at reducing the need for periodontal surgery.

Some wear on the palatal surface of the canine may also be necessary to avoid prematurity and occlusal interference with the mandibular incisors in static and dynamic occlusion. Another aspect that should be foreseen when planning these cases is the possibility of appearance of tooth-size discrepancy, which can affect the quality of intercuspation of these cases. This aspect should be reviewed during the orthodontic finalization phase, and possible mesiodistal adjustments should be performed.^11^ ^,^ ^30^

Ideally, occlusion with balanced group function should be achieved, with distributed occlusal loads and, if necessary, performing an occlusal adjustment for better balance of masticatory forces.^19^

In cases of space opening for placement of endosseous implants in the sites of missing maxillary lateral incisors, it is necessary to evaluate the interradicular space available for implantation. The roots of central incisors and canines should be parallel to each other, providing good working conditions for the implantologist and a favorable biological situation for the responses of peri-implant tissues.

## STABILITY

**Figure 17:**
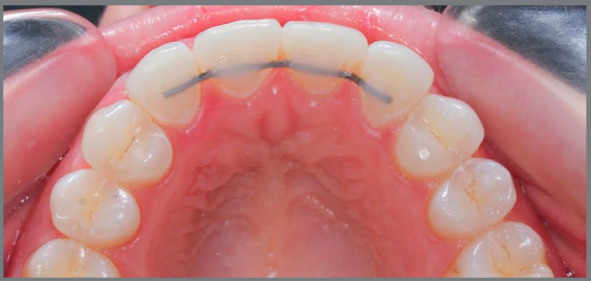
Retainer bonded to central incisors and canines, to increase treatment stability and prevent reopening of small spaces.

Retention is recommended for both treatment options after completion of orthodontics. The tendency of reopening of small spaces is a disadvantage in the treatment with canine positioning in the place of lateral incisor. To avoid such relapse, after restorative esthetic contouring of anterior teeth, the use of a fixed retainer bonded on the palatal aspect of maxillary anterior teeth is recommended (Fig 17). The occlusal balance contemplating the presence of well-balanced disocclusion guidances contributes to avoid or minimize relapse movements. A removable plate is also indicated to complement the retention and protect the teeth in possible para-functions. In some studies, such as conducted by Thordarson and Zachrisson^24^ after a ten-year follow-up, no signs of harmful effects on occlusion were found resulting from the orthodontic treatment of maxillary lateral incisors agenesis.

In cases with space opening, a bridge can be bonded to adjacent teeth until the implant is placed (Fig 4). There is also an indication to use a removable plate to complement the retention. It should be remembered that the inclinations of roots adjacent to the edentulous spaces must be followed radiographically, due to the risk of invading the space left for the implants.^30^

## FINAL CONSIDERATIONS

Attempts to solve the existing controversy about the best treatment option for cases of hypodontia of permanent maxillary lateral incisors would likely be in vain. This issue involves not only orthodontists, but also clinical dentists and prosthodontists, each with their own clinical preferences. The advantages and limitations of both treatment options will always be discussed. Both treatments are long, difficult and expensive.

Regarding one of the main questions, what would be the most conservative treatment since both involve biological costs? Dental wear, to adapt the anatomy and occlusion, and gingivoplasty for esthetic gingival recontouring, in cases of space closure; or dental implant placement surgery and prosthetic reconstruction, in cases of space opening?

Another important controversy is the occlusion resulting from treatment. The literature reveals that there is no superiority of canine guidance over group function. It is worth mentioning the importance of a well-balanced group function in cases of space closure, with disocclusion distributed in the posterior teeth and without interference on the balance side. Poorly completed cases, either with opening or closure of spaces, can trigger abfractions, gingival recessions and joint problems. Premature contacts on the working and balancing side can cause such problems after any type of orthodontic treatment.

Schneider et al. ^30^ concluded in their article that, in the absence of randomized controlled trials on the long-term esthetic and functional stability of standard treatment options for hypodontia of maxillary lateral incisors, the orthodontists, clinical dentists and prosthodontists should avoid imposing their esthetic preferences on the patients.

This statement leads us to reflect on our clinical approach. There is a polarization of radical opinions. Ideally, the orthodontist should have absolute knowledge of the advantages and disadvantages of both treatment options and know how to consider them during diagnosis and planning. All considerations must be exposed to the patient and their caretakers, who must decide on the path to be followed.

Hereby is a reading suggestion for reflection before taking radical positions. Burke and Kelleher wrote an article entitled *“The daughter test”*,^31^ in which they proposed this test in any situation that involves an esthetic and biologically irreversible dilemma, before performing potentially irreversible planning:

“*Knowing what I know about all the factors involved throughout the treatment and the patient’s life, would I do this treatment on my own daughter?*”

Applying this test, perhaps our decision will be easier.
